# N-Acetylcysteine relieving hydrogen peroxide-induced damage in granulosa cells of sheep

**DOI:** 10.1080/19336918.2025.2484182

**Published:** 2025-03-30

**Authors:** Hao Chen, Jine Wang, Bingzhu Zhao, Yahua Yang, Chongfa Yang, Zhijie Zhao, Xiaona Ding, Yang Li, Taojie Zhang, Zhaxi Yingpai, Shengdong Huo

**Affiliations:** College of Life Science and Engineering, Northwest Minzu University, Lanzhou, Gansu, China

**Keywords:** Granulosa cells, N – acetylcysteine, oxidative stress, sheep

## Abstract

Sheep ovarian granulosa cells (GCs) play a unique role in the ovary. Damage to GCs can affect the normal development of oocytes. The oxidative stress model was constructed by H_2_O_2_to study the biological changes. Specifically, pathological characteristic was assessed by immunohistochemistry (IHC), while signaling pathway was studied using western blot, quantitative RT-PCR, and immunofluorescence. The results showed that the oxidative damage model was successfully constructed by 200 μmol/LH_2_O_2_ for 12 h. NAC can protect the proliferation of GCs under H_2_O_2_-induced oxidative stress and reduce apoptosis. It can also promote the secretion of E_2_ and P_4_ by GCs and reduce the inflammatory response of GCs. NAC can enhance the expression of NRF2, PI3K and Akt. These findings suggest that NAC alleviates H_2_O_2_-induced oxidative stress injury through NRF2/PI3K/AKT signaling pathways. Provide ideas for studying the poor quality of mammalian oocytes.

## Highlights

- The molecular mechanism of NAC anti-oxidation through NRF2/HO-1.
- The molecular mechanism of NAC anti-oxidation through PI3K/AKT.
- NAC has antioxidant capacity on granulosa cells.

## Graphical abstract

**Figure:**
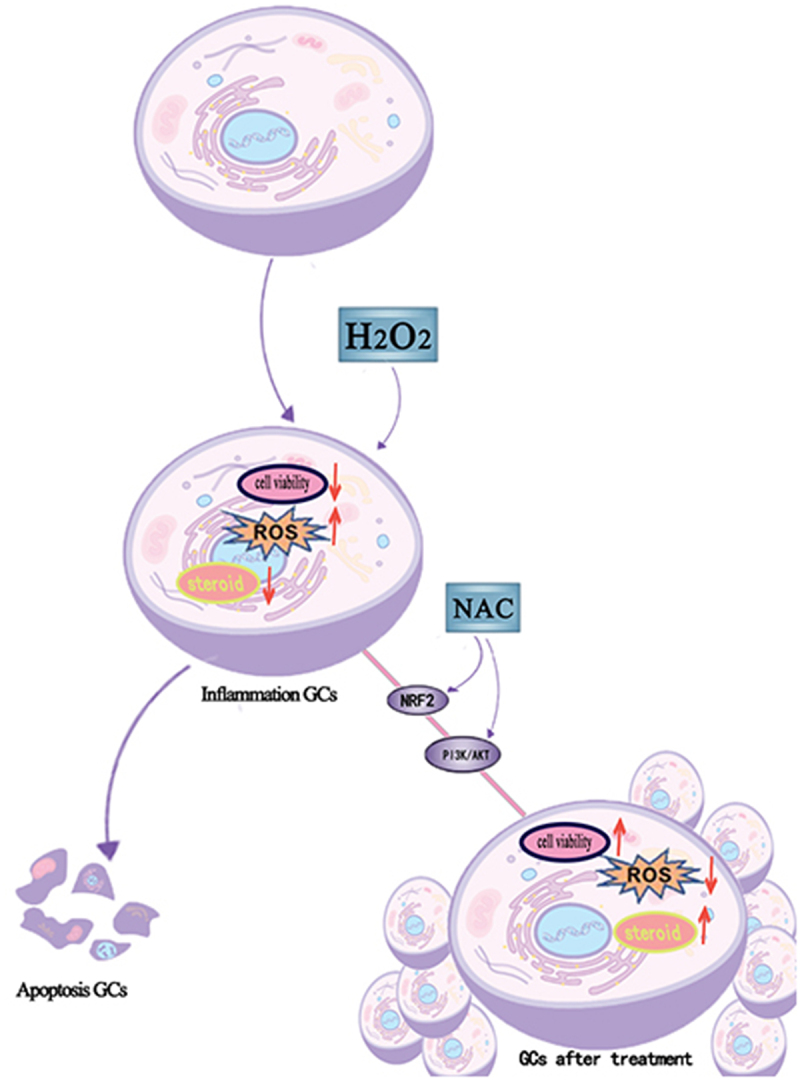

## Introduction

The ovary of mammals is the most dynamic organ in adult females, which is closely related to the reproductive performance of animals. Follicle is the most basic structural and functional unit of mammalian ovary [1]. The mature follicles are composed of oocytes, cumulus cells, ovarian GCs, TCs and follicular fluid. Follicular maturation and ovulation are intricate physiological processes [2]. During these processes, a rapid increase in metabolites can decrease the activity of antioxidant enzymes, leading to elevated levels of reactive oxygen species (ROS) [3]. Under normal conditions, The production and digestion of ROS are transformed into a dynamic equilibrium state, with an appropriate ROS concentration supporting ovarian development and ovulation [4]. However, excessive ROS [5] levels can trigger oxidative stress, promote cell damage, activate apoptosis pathways, and contribute to abnormal follicular atresia, affecting oocyte quality and reproductive outcomes [6].

In mammalian ovaries, GCs proliferate first, and when the number of proliferation reaches a certain level, oocytes begin to develop [7]. In the process of follicular growth and development, there are many regulatory networks between GCs and follicles [8,9]. The proliferation of GCs involves the whole process of follicular growth and development. GCs regulate follicular development by secreting steroid hormones, cytokines, growth factors, etc., providing a good growth environment for follicular development. There is a gap link between GCs and oocytes [7,10]. Through cell gap junctions, oocytes secrete factors such as BMP15 and GDF9 to regulate the proliferation and differentiation of GCs. Through gap junctions, oocytes can also excrete metabolic waste generated inside. GCs can provide and transmit small molecules of nutrients, vitamins, calcium ions, metabolic precursors and signal transduction molecules required for oocytes through gap links. The FSH receptor on the surface of the GCs membrane can bind to FSH, activate the cAMP pathway, promote the expression of aromatase genes, make estrogen synthesized in large quantities, and further promote follicular growth. With the continuous development of follicles, the volume and number of GCs increase. Under the action of LH, GCs undergo luteinization, maintain the secretion of progesterone by luteal cells, and further regulate oocyte maturation and ovulation [11].

When animal cells are cultured in vitro, the appropriate concentration of NAC solution is added to the culture medium, which can increase the proliferation activity of germ cells and inhibit their apoptosis, thus delaying the aging of animals and increasing their life span. That is because N-acetylcysteine (NAC), as an effective antioxidant, can scavenge free radicals in cells and indirectly synthesize reduced glutathione (GSH) [12], thereby mitigating oxidative stress injuries in animals. Studies have demonstrated that NAC can inhibit oxidative stress in various cell cultures and animal models [13].

Therefore, this study aims to investigate the effects of varying concentrations of NAC on GCs proliferation [14], apoptosis, ROS levels, hormone secretion, and related gene and protein expression [15]. By employing the PI3K/AKT inhibitor LY294002 [16], the molecular mechanisms will be explored. Additionally, the relationship between cellular antioxidant capacity and NRF2/PI3K/AKT signaling pathways will be investigated to determine whether NAC alleviates H_2_O_2_-induced oxidative stress damage through these pathways [17].

## Materials and methods

### Cell processing and identification

In this study, the ovaries of healthy ewes aged about 2 years old were isolated and cultured from the slaughterhouse in Wushengyi Town, Gansu Province. Extract follicular fluid from ovarian follicles with a diameter of approximately 3–8 mm into a tube. Filter the follicular fluid through a 100 nm filter, then centrifuge at 1500 r/min to collect the precipitate. The cells were resuspended in DMEM containing 10% fetal bovine serum. Observe cell morphology and culture at 37°C, 5% CO_2_. FSHR immunofluorescence staining was performed when the cell adhesion reached 70%-80%. The cells were fixed in 4% paraformaldehyde at room temperature for 30 min, the immunofluorescence permeabilization solution was used to penetrate the membrane at room temperature for 30 min, and the immunostaining blocking solution was blocked at room temperature for 1 h. Cells were incubated with 1:200 concentration of FSHR at 4°C overnight. The cells were incubated with 1:1000 anti-rabbit secondary antibody at room temperature in the dark for 1 h. Blank control was only incubated with secondary antibody. DAPI staining was performed in dark for 5 min.

### Construction and treatment of GCs oxidative damage model

The primary GCs were inoculated into 96-well plates (density of 1 × 10^4^cells/well), and the blank group was set up:only 100 μL medium was added; control group:90 μL cell suspension and 10 μL culture medium were added; experimental group:90 μL cell suspension was added. The cells were pre-cultured in 5%CO_2_ incubator at 37°C for 12 h, and 10 μL of H_2_O_2_ with final concentrations of 100, 150, 200 and 250 μmol/L was added to the experimental groups, respectively. Then the cells were cultured in 5% CO2 incubator at 37°C for 6, 12, 24 and 36 h, respectively. After that, 10 μL of cck-8 solution was added to each well and incubated for 2 h. The absorbance at 450 nm was measured by microplate reader. When the cell survival rate was as low as about 50%, the determined H_2_O_2_ concentration and induction time were the optimal damage concentration and time for the construction of GCs oxidative damage model. Cells were observed under the microscope to determine the cell status. When the cell confluence reached 80% or more, the cells were digested with 0.25% trypsin and cultured in a 1:2 ratio for passage. The cells were treated with control group, NAC group (100 µmol/L), H_2_O_2_ group (200 µmol/L), and NAC (100 µmol/L) + h_2_O_2_ (200 µmol/L) group. Each group had three biological replicates.

### CCK-8 assay

The cells were seeded in 96-well plates, 1 × 104 cells per well, and the culture medium was discarded after 6 h of culture. The control hole, test hole and blank hole were set up, and NAC solution holes with concentrations of 0,50,100,500 and 1000 μmol/L were added respectively, and only culture medium holes were added, with 3 replicates in each group. Cells in each group were cultured for 24,48 and 72 h, and 10 μL CCK-8 solution was added to each well. After incubation at 37°C for 2 h, the absorbance at 450 nm was measured by microplate reader. Cell proliferation activity was calculated according to the formula. Cell viability (%) = [(A(plus) -A (empty))/(A(0plus) -A(empty))]×100. A(plus):with cells, CCK-8 solution, affecting drugs; a (empty) : with medium, CCK-8 solution, no cells; a (0plus) : with cells, CCK-8 solution, no effect on the drug.

### Flow cytometry

Add 90 μL cell suspension to 96-well plates. After pre-incubation in a 5% CO2 incubator at 37°C for 12 h, 10 μL of H2O2 with final concentrations of 100, 150, 200 and 250 μmol/L was added to the experimental groups, respectively. The cell culture supernatant was collected to centrifuge tubes, and the cells were washed twice with PBS. The stained cell suspension was transferred to a flow tube, and the data were detected and analyzed by flow cytometry.

### ROS determination

The DCHFDA stock solution was diluted with serum-free medium to a final concentration of 10 μM. The cells were slowly washed with PBS for 3 times, then the diluted dye was added. The images were taken under a fluorescence microscope.

### Oxidative stress indicators determination

The levels of glutathione (GSH) and malondialdehyde (MDA), as well as the activity of superoxide dismutase (SOD), were determined using commercial kits. Following treatment, the GCs were collected and washed three times with ice-cold PBS. The resulting pellet was then homogenized with pre-cooled PBS at 4°C or in an ice bath. Subsequently, the homogenate was centrifuged at 10,000 rpm at 4°C for 10 minutes, and the supernatant was collected as the sample for testing. The assays were conducted according to the instructions provided with the GSH, SOD, and MDA detection kits (Biyuntian, Shanghai, China).

### ELISA assay

The culture medium was collected in 1.5 mL centrifuge tubes. The supernatant was obtained after centrifugation at 2500 rpm for 20 minutes. The concentrations were measured by the kits (Shanghai, Mlbio, China). Each group consisted of three biological replicates.

### Immunofluorescence staining

The isolated cells were inoculated into 24-well plates, and the cells adhered to 80%. The cell culture medium was discarded and washed with PBS buffer for 3 times, 2 min each time. Add 250 μL paraformaldehyde to fix for 30 min, discard the fixative, and wash with PBS buffer for 3 times, 2 min each time. Add 250 μL 0.2% Triton X-100, incubate at room temperature for 15 min, discard the liquid and wash with PBS buffer 3 times, 2 min each time. The diluted primary antibody was added to each well, and the same amount of PBS buffer was added to the negative control well. The cells were incubated at 4°C for 12 h, and washed with PBS buffer for 3 times, 5 min each time. Each well was added with 500 μL of diluted secondary antibody and placed in a dark room. After incubation at room temperature for 3 h, it was washed with PBS buffer for 3 times, 5 min each time. In the dark room, 200 μL DAPI staining solution was added to each well, incubated at room temperature for 1 min, and washed with PBS buffer for 3 times, 5 min each time. Observed and photographed by a fluorescence microscope in a dark room.

### RNA isolation, reverse transcription, and quantitative RT-PCR

Total RNA was extracted using the Trizol method (SOLEIBO, Beijing, China), and the concentration and purity of the RNA samples were assessed using a NanoDrop One/OneC spectrophotometer. RNA samples with D260/D280 values between 1.8 and 2.0 were chosen for cDNA synthesis, which was performed using the Prime SCRIPT TMRT kit (Takara, Beijing, China) with 1 μg of high-purity RNA. qRT-PCRwas conducted using SYBR PreMix Ex Taq TM II (Takara, Beijing, China) and a real-time PCR system (Bio-Rad, Hercules, CA, USA). The reaction conditions were as follows: pre-denaturation at 95°C for 3 minutes, followed by denaturation at 95°C for 10 seconds, annealing at 60°C for 30 seconds, and a total of 40 cycles. Each group consisted of three biological replicates. The relative expression was calculated by 2^−ΔΔCt^.

The primers for real-time fluorescence quantitative PCR were designed using Primer Premier 5.0 software based on the sequences of sheep genes available in the GenBank database. The primers were synthesized by Beijing HuadaGene Information Company (Table 1).

**Table 1.** Real-time PCR primer sequences.

Gene	Primer sequence (5′-3′)	Product size/bp	Accession number
*PCNA*	*F-TCTTGAAGAAAGTGCTGGAGGC*	259	XM_004014340.5
	*R-TCCGCATTATCTTCAGCCCTTA*		
*Bcl-2*	*F-ATGCCTTTGTGGAGCTGTATGG*	180	XM_027960877.2
	*R-ACTGAGCAGTGCCTTCAGAGACA*		
*Bax*	*F-GACAGGGGCCCTTTTGCTT*	128	XM_027978592.2
	*R-TCAGACACTCGCTCAGCTTC*		
*CAT*	*F-TTACCAGATACTCCAAGGCGAAG*	221	XM_004016396.5
	*R-AAAGGACGGAAACAGTAGAGCAT*		
*SOD1*	*F-CAATCACAGCATCTTCTGGACAAAT*	215	NM_001280703.1
	*R-CCTGGTTAGAACAAGCAGCAATCT*		
*3β-HSD*	*F-GGAGACATTCTGGATGAGCAG*	209	XM_027961610.2
	*R-TCTATGGTGCTGGTGTGGA*		
*CYP19A1*	*F-ATGCTGGTGCTGAGTATGTGGT*	192	NM_001123000.1
	*R-GCTGACAATCTTGAGGGTGTTG*		
*NRF2*	*F-CACTTCATCTGGCAGCACAGTAT*	238	XM_042252469.1
	*R-ACCACCTCTTGATGATTGTGTTCC*		
*HO-1*	*F-CAGGCCACCAAGTGCTATGT*	141	XM_027967703.2
	*R-CAGGGCCTTCTGAGCAATCT*		
*Keap1*	*F -TGCCCCTGTGGTCAAAGTG* *R -GGTTCGGTTACCGTCCTGC*	104	XM_015274015.1
*ACTIN*	*F-ATATTGCTGCGCTCGTGGTT*	224	NM_001009784.3
	*R-GTTGGTGACAATGCCGTGCT*		

### West-blot assay

Sheep GCs in different treatment groups were washed three times with PBS, and then RIPA cell lysis buffer was mixed with PMSF (100 : 1). Each well was added with 200 μL, lysed on ice for 30 min, centrifuged at 15,000 r/min for 15 min, and the supernatant was collected. After the protein concentration was determined by BCA method, it was placed at-80°C for later use. The protein was mixed with 4 × sodium dodecyl sulfate (SDS) loading buffer (3 : 1) and incubated in a boiling water bath at 100°C for 10 min to prepare protein separation gel (15%) and concentrated gel (5%), respectively. The sample was added to 10 μL protein/lane. After SDS-PAGE electrophoresis, the membrane was electrotransferred to polyvinylidene fluoride (PVDF) membrane, washed once with PBS, blocked with 5% skimmed milk powder solution at room temperature for 3 h, incubated with primary antibody overnight at 4°C, washed three times with PBST, 5 min each time, and added horseradish peroxidase-labeled secondary antibody (1 : 3000). After incubation at 37°C for 1 h, the PVDF membrane was washed three times with PBST for 5 min each time. The electrochemiluminescence solution was added to the PVDF membrane and incubated in the dark for 2 min. The chemiluminescence instrument was used for detection. Finally, the software ImageJ was used to detect the target band.

### Statistical analysis

GraphPad Prism 8 software was used to analysis the data using one-way ANOVA, and the pairwise comparison was performed using the LSD and Tukey’s test. The results were expressed as mean ± standard deviation.

## Results

### Isolation of GCs

**Figure 1.**
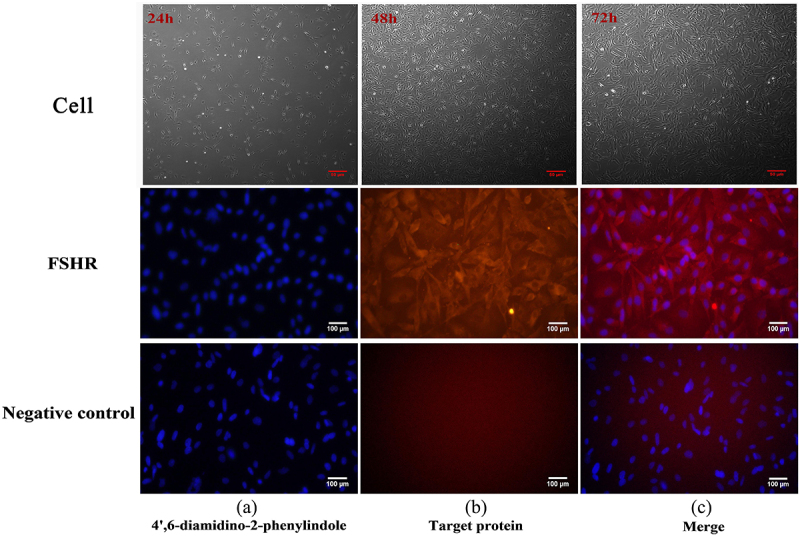
Immunofluorescence images : the growth status of cells at 12 hours, 48 hours, and 72 hours and the immunofluorescence staining images of FSHR.

The primary passage GCs that were cultured for 72 h had fully adherence (Figure 1a–c).

### H_2_O_2_ reduces the viability of GCs cells

**Figure 2.**
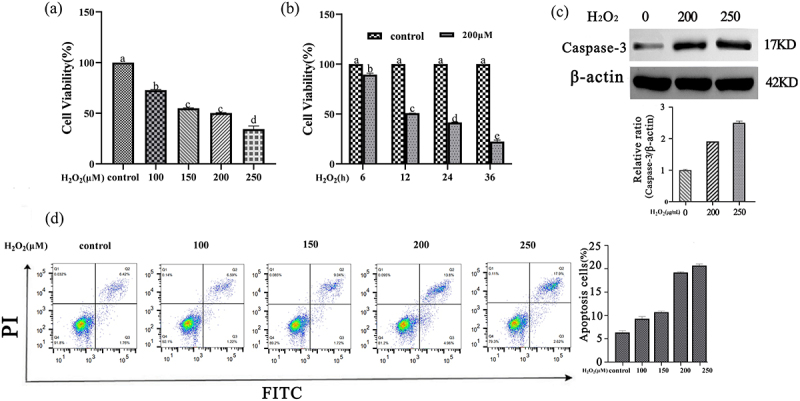
CCK-8, WB and flow cytometry : validation of H2O2-induced GCs apoptosis.

To find the optimal concentration of H_2_O_2_ for cell damage, this experiment treated GCs with different concentrations of H_2_O_2_ for varying durations (Figure 2a,b). Then apoptosis and apoptosis index Caspase-3 were verified by flow cytometry and Western blot (Figure 2c,d). The results showed that a treatment with 200 μM H_2_O_2_ for 12 hours reduced the cell proliferation rate to 50%. Therefore, subsequent experiments all used a treatment of 200 μM H_2_O_2_ for 12 hours on GCs.

### NAC enhance the antioxidant capacity ofH_2_O_2_ onGCs

**Figure 3.**
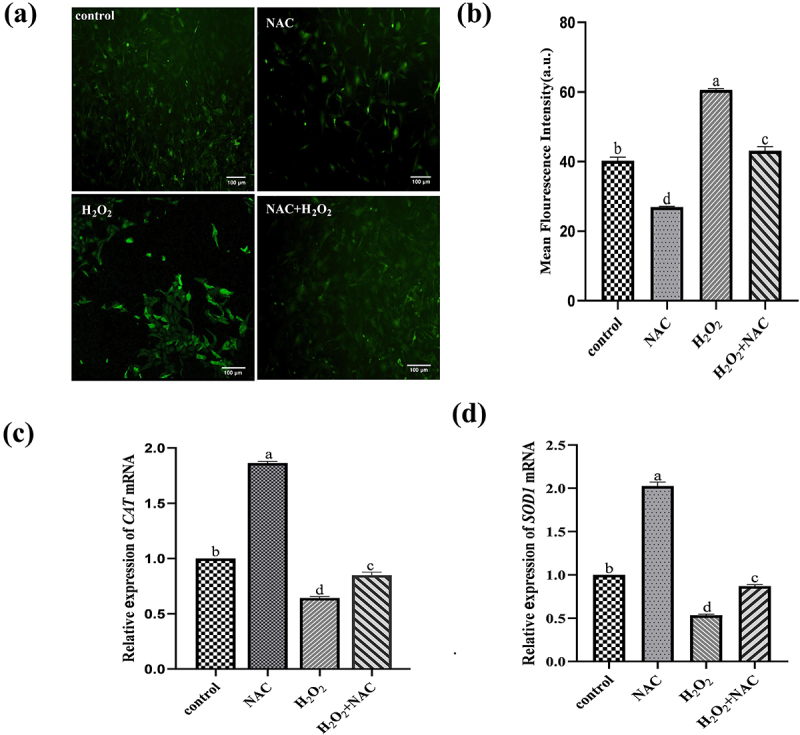
Reactive oxygen species staining and RT-PCR results : the effect of NAC on ROS levels in GCs was evaluated.

The impact of NAC on ROS levels in GCs was evaluated. Compared with the H_2_O_2_ treatment group, the NAC and H_2_O_2_ co-treatment group significantly decreased the levels of ROS (Figure 3a,b). The antioxidant-related gene catalase (*CAT*) and superoxide dismutase 1 (*SOD1*) were assessed by RT-PCR. Compared with the H_2_O_2_ treatment group, the NAC and H_2_O_2_ co-treatment group significantly up-regulated the expression of *CAT* and *SOD1* (Figure 3c,d).

### NAC attenuate the oxidative stress damage ofH_2_O_2_onGCs

**Figure 4.**
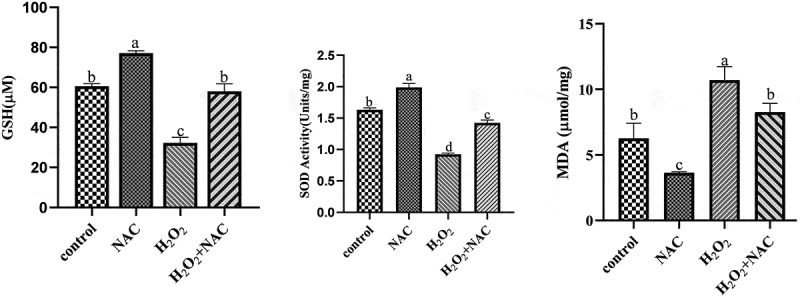
ELISA assay picture: the effect of NAC on oxidative stress indicators of sheep GCs induced by H_2_O_2_was explored.

The effect of NAC on oxidative stress indicators of sheep GCs induced by H_2_O_2_was explored. Compared with the H_2_O_2_ treatment group, the NAC and H_2_O_2_ co-treatment group significantly increased the content of GSH and the activity of SOD (*p* < .01), and decreased the content of MDA (Figure 4).

### NAC inhibit the apoptosis ofH_2_O_2_ onGCs

**Figure 5.**
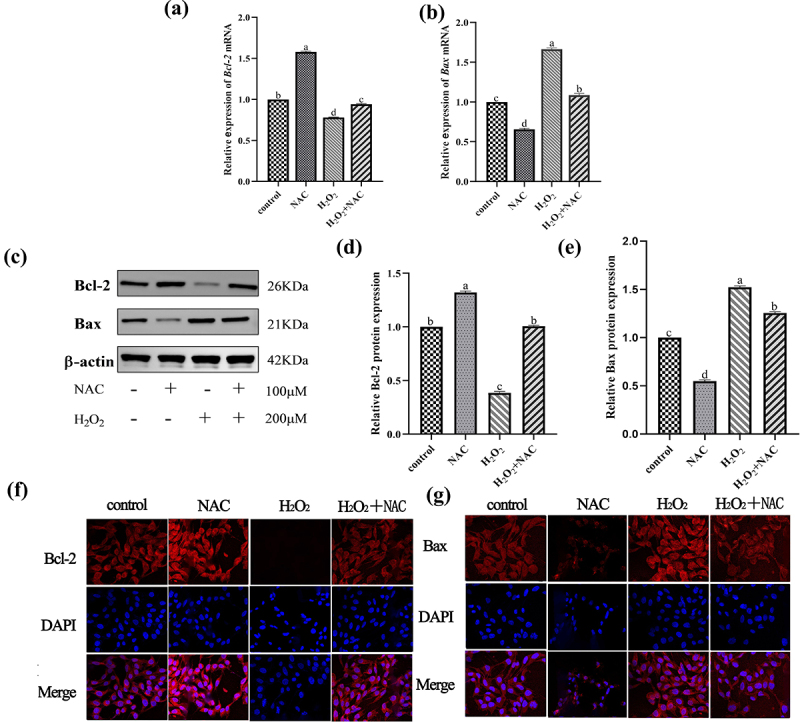
RT-PCR, WB and immunofluorescence images: to verify the effect of NAC on H2O2-induced GCs apoptosis in sheep.

The molecular mechanism of NAC on apoptosis was investigated by measuring the expression levels of apoptosis-related genes (*Bcl-2, Bax*) by qRT-PCR. The results showed that the expression levels of *Bcl-2* were significantly upregulated, *Bax* was significantly down-regulated in the NAC combined with H_2_O_2_ group than in the H_2_O_2_ group (Figure 5a–b). The expression levels of *Bcl-2* and *Bax* proteins were detected by Western blot and immunofluorescence staining (Figure 5c–g). The results showed that *Bcl-2* and *Bax* were consistent with the changes in its genes expression.

### NACcan promote the proliferation of sheep GCs under oxidative stress

**Figure 6.**
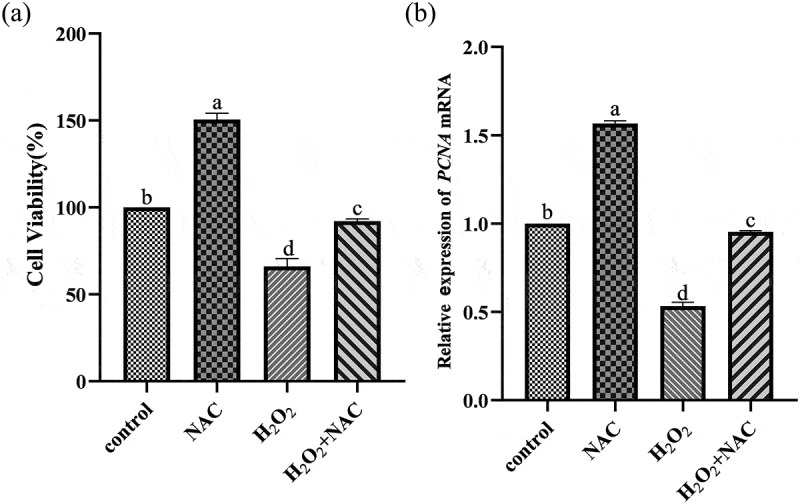
RT-PCR and CCK-8 images: NAC can promote the proliferation of sheep GCs under oxidative stress.

NAC significantly increased the proliferation activity of GCs (Figure 6a) in the NAC combined with H_2_O_2_ group than in the H_2_O_2_ group. The proliferation related genes (*PCNA*) was investigated by qRT-PCR. *PCNA* genes expression levels were significantly up-regulated (Figure 6b) in the NAC combined with H_2_O_2_ group than in the H_2_O_2_ group.

### NAC promote steroid hormone secretion ofH_2_O_2_ onGCs

**Figure 7.**
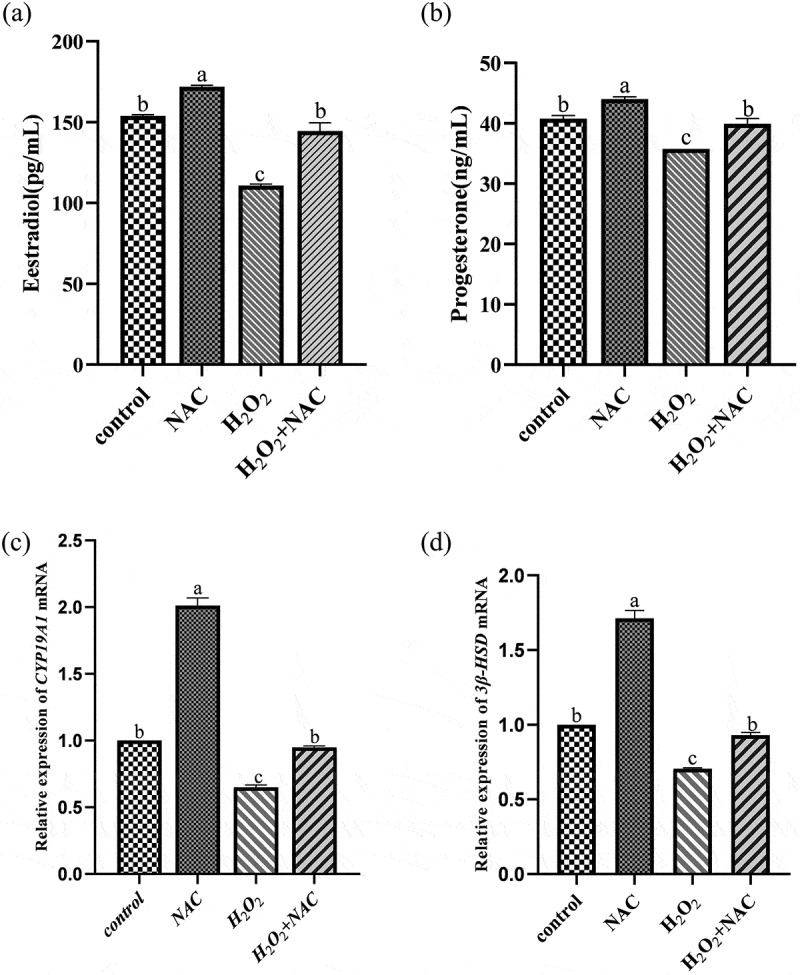
ELISA and qRT-pcr results: NAC promoted steroid hormone secretion of H2O2 on GCs.

The level of steroid hormone secretion was assessed by ELISA for progesterone and androgen content in the culture medium supernatant. The secretion of E_2_ and P_4_-were increased significantly (Figure 7a–b) in the NAC combined with H_2_O_2_ group than in the H_2_O_2_ group. The molecular mechanism was investigated by measuring the expression levels of steroidogenesis-related genes (*CYP19A1*and *3β-HSD*) by qRT-PCR. The results showed that the *CYP19A1*and *3β-HSD* were significantly upregulated (Figure 7c–d) in the NAC combined with H_2_O_2_ group than in the H_2_O_2_ group.

### NAC inhibit inflammation ofH_2_O_2_onGCs

**Figure 8.**
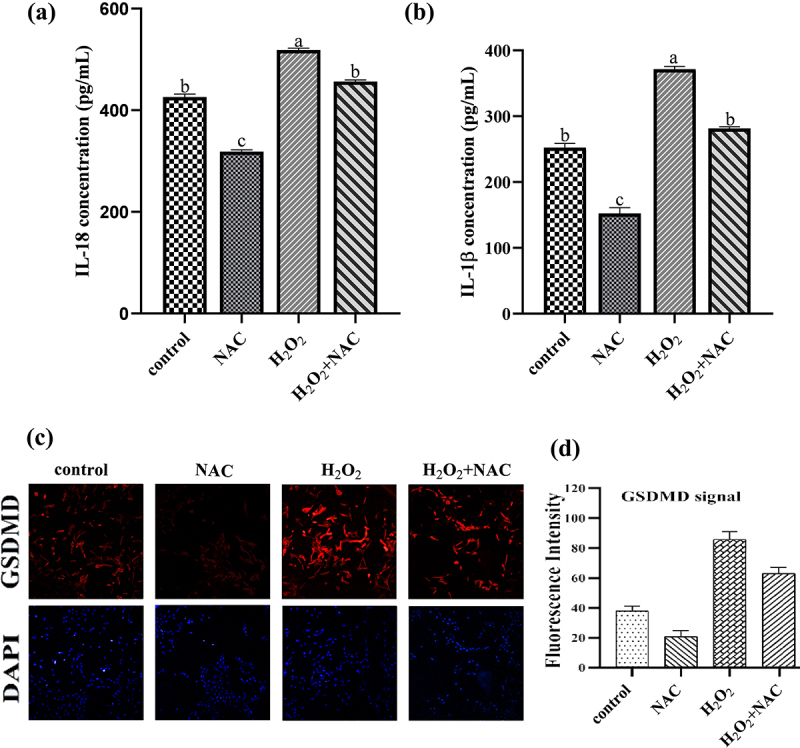
Results of ELISA and immunofluorescence: NAC inhibited the inflammation of H2O2 on GCs.

ELISA and immunofluorescence staining showed that NAC combined with H_2_O_2_ significantly reduced IL-18, IL-1 and GSDMD in the supernatant (*p* < .05) (Figure 8a–d).

### NAC alleviates ofH_2_O_2_onGCs through NRF2/HO-1 and PI3K/AKT signal pathways

**Figure 9.**
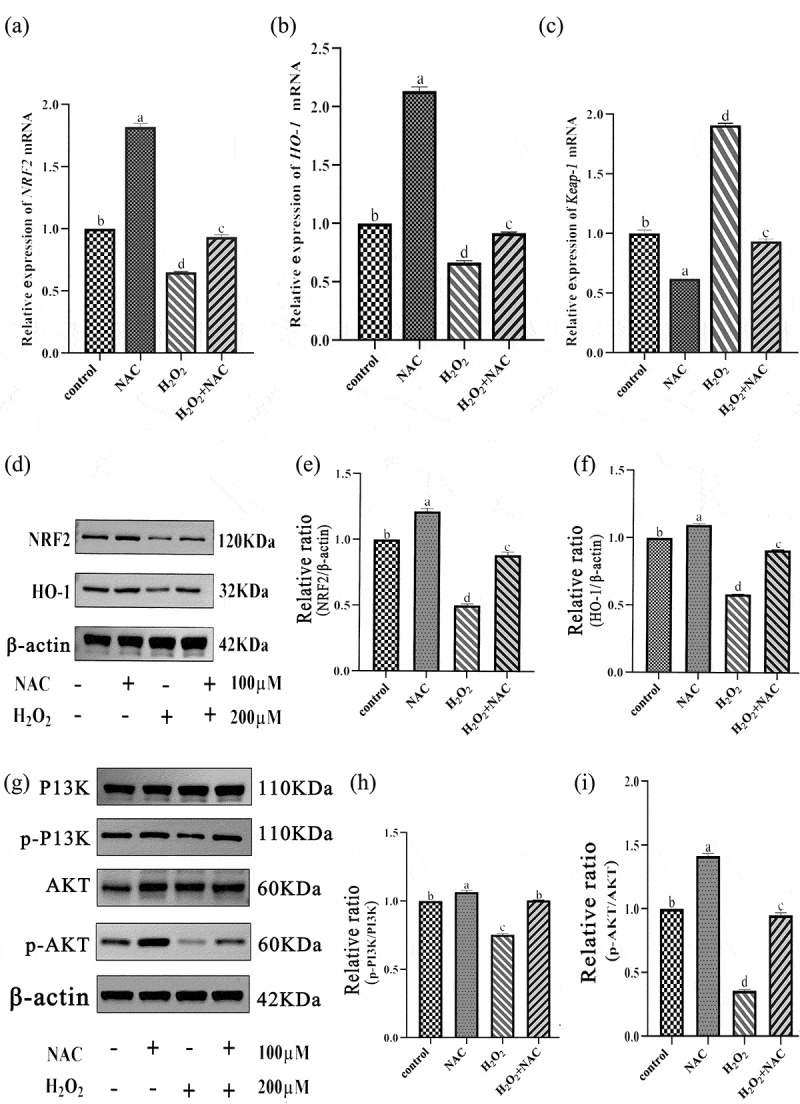
Results of qRT-PCR and WB: to verify the effect of NAC on H2O2-induced GCs antioxidant signaling pathway.

Compared with NAC and H_2_O_2_ treatment groups, NAC and H_2_O_2_ co-treatment groups significantly up-regulated the mRNA levels of *NRF2* and *HO-1* genes and down-regulated the mRNA level of *Keap-1* gene (Figure 9a–c). Compared with NAC and H_2_O_2_ groups, the expression levels of *NRF2* and *HO-1* protein in NAC and H_2_O_2_ co-treatment groups were significantly increased (*p* < .05) (Figure 9d–f). Compared with NAC and H_2_O_2_ groups, the expression levels of p-PI3K/PI3K and p-Akt/Akt in NAC and H_2_O_2_ co-treatment groups were significantly increased (*p* < .05) (Figure 9g–i).

## Discussion

Female mammals experience stress from lactation and pregnancy, which can lead to excessive production of ROS [18], primarily including superoxide anion radical (O^2-^), hydroxyl radical (OH^−^), and H_2_O_2_. Oxidative stress adversely affects the health and reproductive performance of female animals [19]. Among various causes of reproductive disorders in sheep, H_2_O_2_ is a key factor in inducing oxidative stress [20]. It is crucial to explore oxidative stress and the mechanisms related to H_2_O_2_, a reactive oxygen species molecule that can increase intracellular ROS and cause cellular damage [21]. H_2_O_2_ is commonly used to quickly establish cell oxidative stress injury models.

Cell proliferative activity is an essential indicator for assessing the in vitro proliferation capacity of cells under different treatment conditions [22]. In this study, 200 μmol/L H_2_O_2_ treatment for 12 hours resulted in a cell survival rate of 50.29%, indicating that GCs were somewhat damaged but retained some proliferative and differentiative abilities. Therefore, this concentration and duration of H_2_O_2_ exposure were chosen to construct the GCs oxidative damage model.

Oxidative stress can impair follicular GC function, leading to poor oocyte quality and reduced reproductive performance in mammals [23]. H_2_O_2_ treatment increased ROS levels in GCs [24], elevated malondialdehyde (MDA) content, and decreased glutathione (GSH) content and SOD activity. N-acetylcysteine (NAC), an antioxidant, can mitigate H_2_O_2_ effects on GCs by scavenging ROS, reducing MDA content, and increasing SOD activity [25]. Studies have demonstrated that NAC can inhibit oxidative stress-induced apoptosis in various cell types, promote oocyte maturation, and improve early embryonic development by modulating ROS and GSH levels [26].

During follicular development, the proliferation of GCs is crucial for oocyte growth and ovarian function [27]. H_2_O_2_ treatment significantly inhibited GC proliferation and induced apoptosis, while NAC pretreatment significantly mitigated these adverse effects. NAC may promote GC proliferation by increasing the level of the *PCNA* gene, a marker of cell proliferation and important for cell cycle regulation [28].

Furthermore, quantitative RT-PCR showed that NAC treatment significantly upregulated the anti-apoptotic gene *Bcl-2* and downregulated the pro-apoptotic gene *Bax*. GC apoptosis is a key factor in follicular atresia, a selective process in mammalian folliculogenesis. NAC has been shown to attenuate oxidative stress-induced apoptosis by modulating pathways such as *Bax*/Caspase-3 [29].

In mammalian follicular development, GCs surround oocytes, providing nutrition and secreting hormones. H_2_O_2_ treatment significantly reduced the secretion of P_4_ and E_2_ by GCs, but NAC pretreatment followed by H_2_O_2_ exposure significantly increased P_4_ and E_2_ secretion. The expression of steroidogenesis-related genes *CYP19A1* and *3β-HSD* was also upregulated by NAC pretreatment, indicating a positive effect of NAC on steroid hormone secretion by sheep GCs under oxidative stress [30].

Oxidative stress can promote inflammation. NAC has been shown to inhibit apoptosis and inflammatory responses induced by various stressors in different cell types and to improve inflammatory conditions. In this experiment, the levels of inflammatory cytokines IL-18 and IL-1β increased with H_2_O_2_ treatment but significantly decreased after NAC pretreatment and H_2_O_2_ exposure, suggesting that NAC can effectively reduce the levels of these inflammatory factors in an oxidative stress model [31].

NRF2 is involved in the cellular response to oxidative stress, and it signaling pathway regulates metabolic and antioxidant enzymes. Under oxidative stress, NRF2 dissociates from its inhibitor Keap1 and translocates to the nucleus to regulate the transcription of antioxidant genes such as *HO-1*. The phosphatidylinositol 3-kinase (PI3K)/Akt pathway is an upstream signal of NRF2and plays a protective role against oxidative stress. NAC has been shown to activate the PI3K/Akt signaling pathway, which in turn activates NRF2/HO-1 signaling, protecting cells from apoptosis and improving oocyte quality.

## In conclusion

The oxidative damage model was successfully constructed by 200 μmol/L H_2_O_2_ for 12 h. It was found that NAC could protect the proliferation of GCs and reduce the content of ROS and MDA under H_2_O_2_-induced oxidative stress. Increase GSH content and SOD activity. Reduce cell apoptosis and inflammatory factors IL-18, IL-1 content; up-regulate the protein of *Bcl-2*, down-regulate the expression level of Baxgene and protein; inhibition of NRF2 and HO-1 gene and protein expression; promote the secretion of E_2_ and P_4_ by granulosa cells. The molecular mechanism may be that NAC alleviates H_2_O_2_-induced oxidative stress injury through NRF2 and PI3K/AKT signaling pathways.

## Supplementary Material

Graphical summary.jpg

## Data Availability

The datasets used and/or analyzed during the current study are available from the corresponding author on reasonable request.

## Author statement

All authors have read and approved the final version of the manuscript.
