# Supplementary figures and images for: N-Acetylcysteine relieving hydrogen peroxide-induced damage in granulosa cells of sheep
Source: Cell Adh Migr. 2025 Mar 30;19(1):2484182. doi: 10.1080/19336918.2025.2484182 (PMC11959897; doi:10.1080/19336918.2025.2484182)

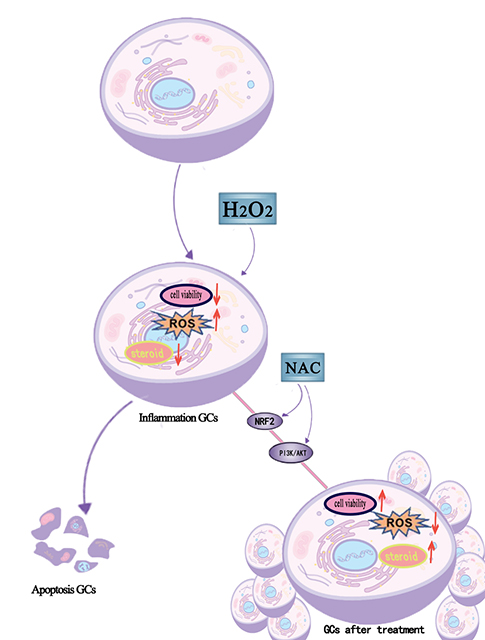

Supplement: Graphical summary.jpg [file KCAM_A_2484182_SM8582.jpg]
